# Experimental Demonstration of Secure Relay in Quantum Secure Direct Communication Network

**DOI:** 10.3390/e25111548

**Published:** 2023-11-16

**Authors:** Min Wang, Wei Zhang, Jianxing Guo, Xiaotian Song, Guilu Long

**Affiliations:** 1 Beijing Academy of Quantum Information Sciences, Beijing 100193, China; wangmin@baqis.ac.cn (M.W.);; 2 State Key Laboratory of Low-Dimensional Quantum Physics and Department of Physics, Tsinghua University, Beijing 100084, China; 3 Frontier Science Center for Quantum Information, Beijing 100084, China; 4 Beijing National Research Center for Information Science and Technology, Beijing 100084, China

**Keywords:** quantum secure direct communication, quantum network, post-quantum cryptography, secure relay

## Abstract

Quantum secure direct communication (QSDC) offers a practical way to realize a quantum network which can transmit information securely and reliably. Practical quantum networks are hindered by the unavailability of quantum relays. To overcome this limitation, a proposal has been made to transmit the messages encrypted with classical cryptography, such as post-quantum algorithms, between intermediate nodes of the network, where encrypted messages in quantum states are read out in classical bits, and sent to the next node using QSDC. In this paper, we report a real-time demonstration of a computationally secure relay for a quantum secure direct communication network. We have chosen CRYSTALS-KYBER which has been standardized by the National Institute of Standards and Technology to encrypt the messages for transmission of the QSDC system. The quantum bit error rate of the relay system is typically below the security threshold. Our relay can support a QSDC communication rate of 2.5 kb/s within a 4 ms time delay. The experimental demonstration shows the feasibility of constructing a large-scale quantum network in the near future.

## 1. Introduction

In traditional communication networks, secret messages are encrypted with classical cryptosystems such as the advanced encryption standard (AES) or the RSA algorithm. As an important part of the communication network, the relays receive ciphertexts from the previous sender and send the reproduced signals to the next node without decrypting the message. Only the intended receiver at the end node recovers the messages with their secret key. Hence, relays are indispensable for the expansion of the communication network.

Recently, the fast development of quantum technology has increased concerns about security and privacy in our communication networks [1,2]. To avoid compromising the security of information, efforts have been devoted to two scientific fields, namely, quantum cryptography [3] and post-quantum cryptography (PQC) [4]. Quantum cryptography exploits the quantum principles to design new protocols, such as quantum key distribution (QKD) [3,5,6], quantum secret sharing (QSS) [7], and quantum secure direct communication (QSDC) [8,9]. QKD enables a secure private key establishment between legitimate parties over a quantum channel. QSDC securely transmits secret messages with quantum states [10,11,12,13,14,15,16,17,18,19]. Post-quantum cryptography [4] ensures the security of public-key cryptographic algorithms by some carefully chosen problems which are computationally hard even with quantum computer.s Recently, the National Institute of Standards and Technology (NIST) selected CRYSTALS-KYBER [20,21] as the public-key encryption and key-establishment algorithm to be standardized.

Due to the lack of quantum relays, quantum networks are presently realized in restricted areas, such as the star-type quantum network [22,23]. At present, trusted relays are utilized to extend the quantum network into a large area. However, these requires means other than cryptography to guarantee their security. They cannot be used directly in existing networks. In Ref. [24], the combination of quantum cryptography with the post-quantum algorithm was proposed, offering a practical way to realize a quantum network with end-to-end computational security.

In this paper, we demonstrate a real-time QSDC network with computationally secure relay by applying CRYSTALS-KYBER to encrypt the message for transmission of QSDC. The intermediate nodes can only receive the ciphertext of the message. The intended receiver uncovers the secret message with the private key after a series of QSDC communications. The time delay and communication rate, two important performance indicators, are measured. The results demonstrate the feasibility of the computationally secure relay, which offers a practical way to build a large-scale quantum network.

## 2. Experiment

**Figure 1:**
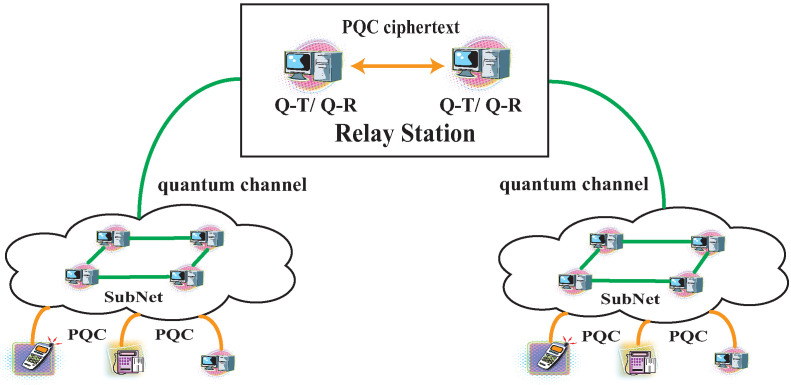
The architecture of QSDC network with computationally secure relay. The orange line denotes the classical channel. The green line denotes the quantum channel. PQC: post-quantum cryptography; Q-T: quantum transmitter; Q-R: quantum receiver.

The schematic of the QSDC network is shown in Figure 1. It can support communication for local-area and large-scale networks. For users of the subnetwork, they can implement QSDC directly. The feasibility of QSDC has been demonstrated by several experiments [11,12,13,18]. Here, we focus on the experimental study of the large-scale QSDC network. It requires a secure relay to build connections between the transmitter and the receiver.

**Figure 2:**
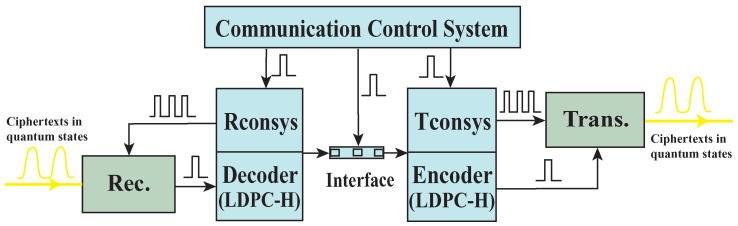
The architecture of the computationally secure relay. Rec.: receiver; Rconsys: receiver control system; Trans.: transmitter; Tconsys: transmitter control system; LDPC-H: low-density parity check and Hadamard code.

The architecture of the computationally secure relay is shown in Figure 2. The control systems (Rconsys and Tconsys) offer the calibration and compensation signals for the receiver and the transmitter. The ciphertexts are transmitted to the encoder after the quantum signals are detected and decoded. The system clock of the relay is controlled by the communication control system.

With the help of the secure relay, our QSDC network can offer services to classical apparatus such as telephones of the user. The signals from the phone are encrypted using the post-quantum algorithm [4]. We then use the QSDC system to transmit the ciphertexts coded in the quantum state. The intermediate node receives the quantum signals and decodes them to recover the ciphertexts. Due to the lack of a private key, the intermediate nodes cannot decrypt the ciphertexts, even with quantum computers. Then, the ciphertexts are transmitted to the next node in the same manner until they reach the intended receiver. The receiver decrypts the ciphertexts with their private key and reads the message. Apparently, our QSDC network has end-to-end security. If the QSDC transmissions are replaced by classical communication, the network is just an example of a classical network as currently in use. The use of QSDC enhances the quantum security mechanically, because eavesdroppers cannot steal the ciphertexts in the transmission, and are confined to only the relay nodes. The ciphertexts are protected by PQC.

**Figure 3:**
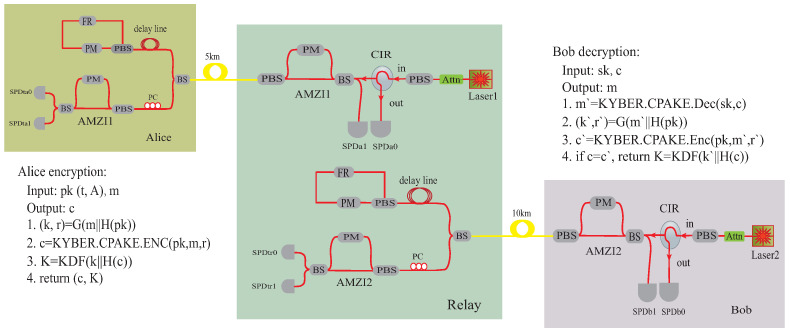
Experimental setup of the QSDC network with a computationally secure relay. Alice encrypts the message and sends the ciphertext to the relay through the quantum channel. Bob receives the signals from the relay and decrypts the message after implementing the QSDC protocol. KYBER makes use of two hash functions H (SHA3-256) and G (SHA3-512) and a key derivation function KDF (SHAKE-256) in the realization. AMZI: asymmetric Mach–Zehnder interferometer; Attn: attenuator; PC: polarization controller; PM: phase modulator; BS: beam splitter; PBS: polarization beam splitter; CIR: circulator; FR: Faraday rotator; SPD: single-photon detector.

To show the feasibility of the QSDC network, we make a real-time experimental test of the computationally secure relay. The experimental setup is shown in Figure 3. Since NIST has recommended CRYSTALS-KYBER as the key-establishment algorithm to be standardized [4], we choose it in our experiment. CRYSTALS-KYBER [20,21] is an indistinguishability under chosen ciphertext attack (IND-CCA) secure key-encapsulation mechanism (KEM). The construction of KYBER follows a two-stage approach: first design an indistinguishability under chosen plaintext attack (IND-CPA) secure public-key encryption scheme, then use Fujisaki–Okamoto (FO) transform to construct the IND-CCA KEM. The security of KYBER is based on the hardness of solving the learning-with-errors problem in module lattices (MLWE), which is believed to be computationally hard to solve even with quantum computers. At the beginning of the experiment, we implement the algorithm of CRYSTALS-KYBER to generate the public key and private key. The public key is published to every participant of the network. The private key is only known to Bob. In our experiment, we choose the parameter set of KYBER-512, which can offer 107 bits of core-SVP quantum hardness [20,21].

We take advantage of the plug-and-play architecture to implement the DL04 protocol [9,14]. The DL04 protocol contains four steps. First, the legitimate receiver prepares a sequence of qubits randomly in one of four states, namely, |0〉 or |1〉 for the Z basis, |+〉 or |−〉 for the X basis. Then, they send the prepared qubits to the transmitter. Second, the transmitter randomly chooses some of the qubits and measures them in the Z basis or the X basis randomly. Then, they communicate with the legitimate receiver to estimate the qubit error rate (QBER) of the quantum channel. Third, if the QBER is typically below the security threshold, the transmitter encodes the message with the remaining qubits by taking advantage of the low-density parity check and Hadamard code (LDPC-H). The encoded qubits are sent back to the legitimate receiver. Finally, the legitimate receiver decodes the message from the received qubits when the error rate is below the correcting capability of the LDPC-H code.

In the experiment, Alice and Bob are each connected to the computationally secure relay through independent 5 km and 10 km spooled standard fiber links. Alice uses the public key of KYBER-512 to encrypt the message. After receiving the optical pulses from the relay, Alice takes advantage of two single-photon detectors (SPDta0 and SPDta1) to monitor the quantum channel. If the quantum bit error rates are typically below the security threshold, Alice encodes the ciphertext into quantum states and returns the quantum signals to the relay. The relay takes advantage of SPDa0 and SPDa1 to decode the quantum signals. In the case of the private key not being available, the relay cannot decrypt the ciphertext. Thus, the security of the message is well protected by KYBER. Then, the relay communicates with Bob in the same manner. SPDtr0 and SPDtr1 are used to monitor the QBERs of the quantum channel between Bob and the relay. After Bob receives the quantum signals, he decodes them with SPDb0 and SPDb1. Finally, Bob decrypts the ciphertext to recover the message with his private key of KYBER.

## 3. Results

The QSDC network works at a repetition rate of 16 MHz. For the SPDs, we choose the efficiency to be 10% with a gate width of 1 ns. The dark count rate is about $1\times 10^{-6}$ per gate. The encrypted messages are transmitted through the quantum channel from Alice, to the secure relay, and then to Bob. The delay time at the secure relay is defined as the time interval between the successful reception of the encrypted messages from Alice and the successful transmission of the encrypted messages to Bob.

**Figure 4:**
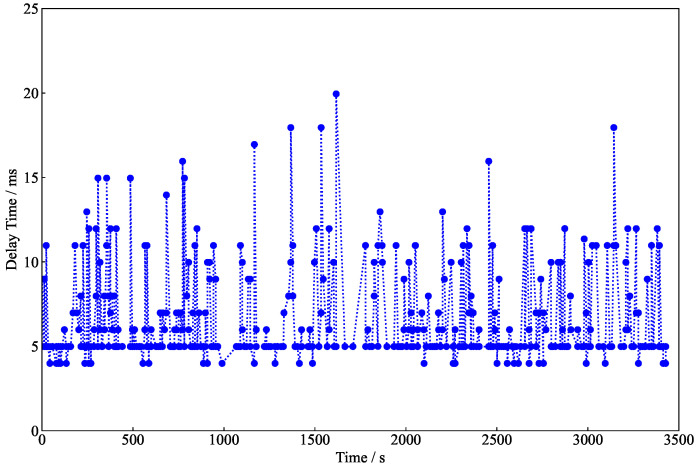
Delay time at the relay.

As shown in Figure 4, the minimum delay time can reach 3.99 ms, which indicates smooth transmission at the secure relay. However, due to noise in the quantum channel, some messages fail to be decoded. In this case, the encrypted messages are sent again, which causes a larger delay time at the secure relay.

**Figure 5:**
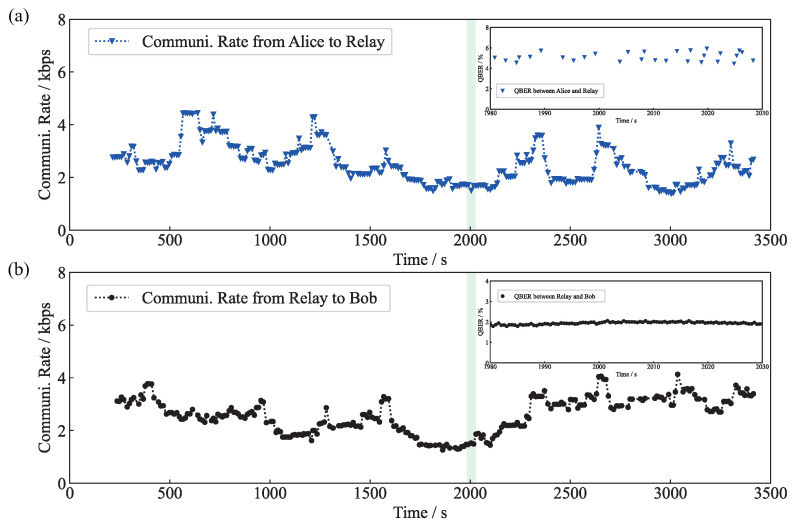
Communication rate of the relayed QSDC system. Communication rate (**a**) from Alice to relay; (**b**) from relay to Bob. Insets are the typical QBER of the QSDC system.

In the experiment, two sets of SPDs are used to monitor the QBERs, namely, SPDta0 and SPDta1 monitor the QBERs of the quantum channel between Alice and the relay, and SPDtr0 and SPDtr1 monitor the QBERs of the quantum channel between the relay and Bob. The QBERs are below the error rate threshold, which ensures the security of our QSDC network. The ciphertexts are recovered with the error-correcting code. As shown in Figure 5, the communication rate between Alice and the secure relay is about 2.5 kb/s, and the communication rate between the secure relay and Bob is about 2.6 kb/s. The overall communication rate of the secure relay system is determined by the lower rate. Therefore, the secure relay can support a QSDC communication rate of 2.5 kb/s within a 4 ms time delay.

## 4. Conclusions

In conclusion, we have realized a real-time demonstration of a secure relay for the QSDC network. The post-quantum algorithm CRYSTALS-KYBER is used to protect the security of the message at intermediate nodes. Only the intended receiver can decrypt the ciphertexts. The results pave the way to realizing a practical QSDC network using existing technology.

It is worth noting that the performance of the QSDC network can be significantly improved further by applying several optimizations. First, it is possible to use a QSDC system with fiber links of over 100 km with the masking technology and superconducting nanowire single-photon detectors (SNSPDs). Second, we take advantage of the software implementation of KYBER directly in our experiment. It is believed that the hardware implementation can further optimize the efficiency.

Our work provides a solution to construct large-scale quantum networks using secure relays. On the other hand, since NIST has announced the standardization of post-quantum algorithms, we can see the widespread application of PQC in communication networks in the future. However, the PQC algorithm may face store-now-decrypt-later (SNDL) [25] attacks. The QSDC transmission of the ciphertexts prevents eavesdroppers from obtaining them in the communication, thus reduces the risks.

## Data Availability

Data are contained within the article.
